# A child with polyarthritis and chronic lung disease: a case report of ataxia-telangiectasia

**DOI:** 10.1186/s13052-023-01509-5

**Published:** 2023-09-04

**Authors:** Laura De Nardi, Marco Francesco Natale, Virginia Messia, Paolo Tomà, Fabrizio De Benedetti, Antonella Insalaco

**Affiliations:** 1 University of Trieste, Piazzale Europa 1, Trieste, 34127 Italy; 2 Bambino Gesù Children’s Hospital, IRCCS, Rome, Italy

**Keywords:** Ataxia-telangiectasia, Juvenile idiopathic arthritis, Bronchiectasis, Interstitial lung disease, Granulomatosis, Immunodeficiency, Case report.

## Abstract

**Background:**

Ataxia-telangiectasia (A-T) is a rare autosomal recessive DNA repair disorder, characterized by progressive cerebellar degeneration, telangiectasia, immunodeficiency, recurrent sinopulmonary infections, radiation sensitivity, premature aging and predisposition to cancer. Although the association with autoimmune and chronic inflammatory conditions such as vitiligo, thrombocytopenia and arthritis has occasionally been reported, an onset with articular involvement at presentation is rare.

**Case presentation:**

We herein report the case of a 7-year-old Caucasian girl who was admitted to the Rheumatology Department with a history of febrile chough and polyarthritis which led initially to the suspicion of an autoinflammatory disease. She had overt polyarthritis with knees deformities and presented with severe pneumonia. A chest Computed Tomography (CT) scan showed bilateral bronchiectasis, parenchymal consolidation and interstitial lung disease; rheumatoid factor and type I interferon signature resulted negative, therefore excluding COatomer Protein subunit Alpha (COPA) syndrome. A diagnosis of sarcoidosis had been suspected based on histological evidence of granulomatous liver inflammation, but ruled out after detecting normal angiotensin converting enzyme and chitotriosidase blood levels. Based on her past medical history characterized by at least six episodes of pneumonia in the previous 4 years, immunological phenotyping was performed. This showed complete IgA and IgE deficiency with defective antigen-specific antibodies to Pneumococcal, Tetanus toxin and Hemophilus Influenzae B vaccines. Additionally, low numbers of B cells and recent thymic emigrants (RTE) were found (CD4Ra 1.4%), along with a low CD4+/CD8 + T cells ratio (< 1). Finally, based on gait disturbances (wobbly wide-based walking), serum alfa-fetoprotein was dosed, which resulted increased at 276 ng/ml (normal value < 7 ng/ml). A diagnosis of Ataxia-Telangiectasia was made, strengthened by the presence of bulbar telangiectasia, and then confirmed by Whole Exome Sequencing (WES).

**Conclusions:**

Although rare, A-T should always be ruled out in case of pulmonary bronchiectasis and gait disturbances even in the absence of bulbar or skin telangiectasia. Autoimmune and granulomatous disorders must to be considered as differential diagnosis.

**Supplementary Information:**

The online version contains supplementary material available at 10.1186/s13052-023-01509-5.

## Background

Ataxia-telangiectasia (A-T) is an autosomal recessive genomic instability syndrome, resulting from a mutation of the Ataxia-Telangiectasia Mutated (ATM) gene encoding for the ATM kinase. The ATM kinase is involved in maintaining cell-cycle homeostasis and in coordinating the cellular response to DNA double-strand breaks and to genotoxic and oxidative stress [1]. Given the pleiotropic role of such a kinase, the phenotype of the disease is unsurprisingly complex and heterogeneous, characterized by neurodegeneration with progressive cerebellar ataxia, postural instability, choreoathetosis, oculomotor apraxia, oculocutaneous telangiectasia and hormonal dysfunction with growth retardation, insulin-resistant diabetogenic response and premature aging [1]. Affected patients also show combined humoral and cellular immunodeficiency with immune dysregulation, predisposition to lymphoid malignancies and sensitivity to ionizing radiation which are markers of chromosomal instability. The great inter-individual variability and age differences in the severity of each single manifestation may sometimes be misleading for clinicians, especially when the onset occurs with non-classical features. Although some associations with autoimmune diseases have been reported, with vitiligo, thrombocytopenia and arthritis being the most frequent [1], an onset with early articular involvement is extremely unusual.

## Case presentation

**Fig. 1:**
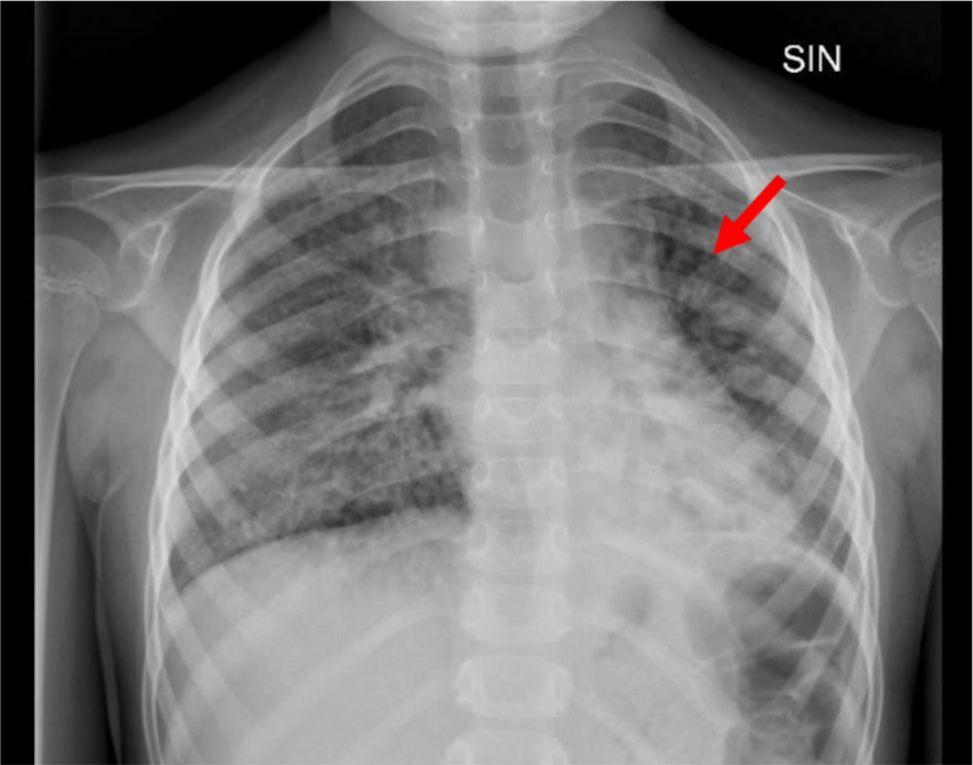
Chest X-ray. Retrocardiac pulmonary consolidation in the left lower lobe is detectable. Bilateral bronchiectasis (red arrow) and interstitial parabronchial enhancement are also evident

**Fig. 2:**
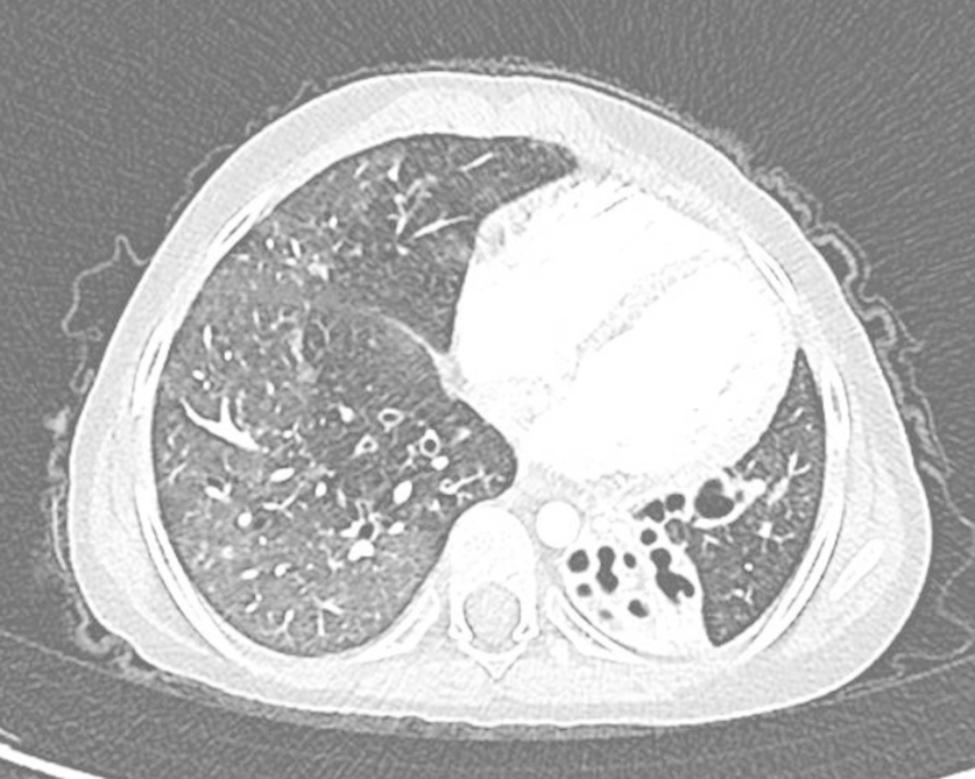
Chest CT scan showing retrocardiac lower lobe consolidation with bronchiectasis. Unilateral hemithorax hyperlucency is detectable, also known as Swyer-James-MacLeod syndrome (SJS), result of postinfectious obliterative bronchiolitis [21]

**Fig. 3:**
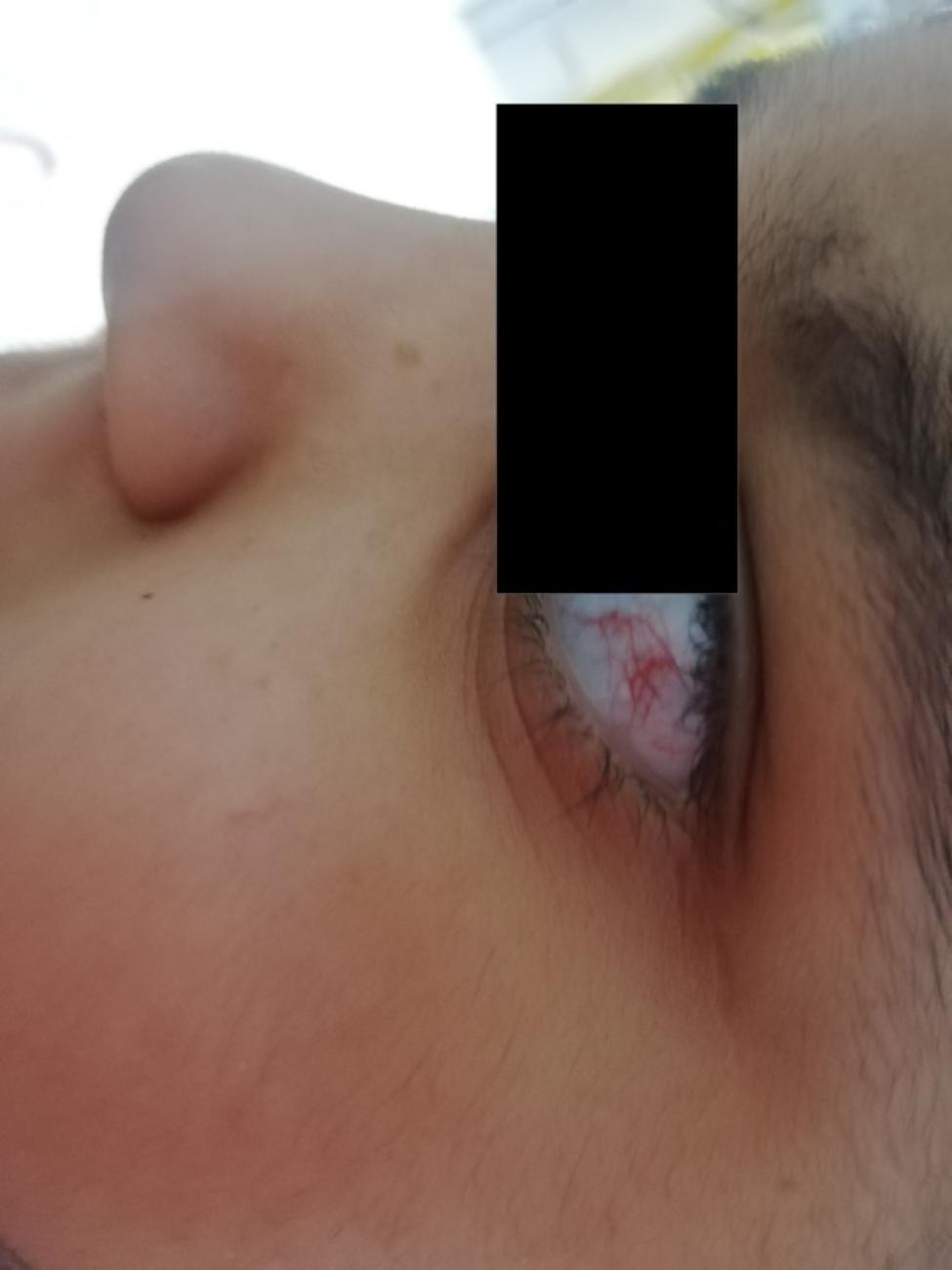
Bulbar telangiectasia

A 7-year-old Caucasian girl, born from non-consanguineous parents, was admitted to the Rheumatology Department of “Bambino Gesú” Children Hospital for a 5 days-history of febrile cough and chronic polyarthritis referred to as present for years. Her physical examination was remarkable for reduction of left basal breath sound and disseminated crackles, as well as for swelling and flexion deformities of both knees and wrists. At admission, complete blood cell count showed neutrophilic leukocytosis and thrombocytosis (white blood cells 40.000/µL, neutrophils 34.000/µL, lymphocytes 3720/µL, platelets 700.000/µL, hemoglobin 11.6 g/dl), with increase in C-reactive protein (180 mg/L). Blood chemistry also showed increase in alanine aminotransferase (ALT 109 U/L) and γ-glutamyl transferase (GGT 203 U/L) levels. Chest X-ray revealed a retrocardiac left basal consolidation, with bronchiectasis images (Fig. 1). A chest CT scan showed a significant pulmonary impairment characterized by a left basal lobe consolidation, bilateral bronchiectasis and interstitial lung disease (ILD) (Fig. 2). The girl was treated with intravenous ceftriaxone with prompt resolution of the respiratory picture. Her past medical history revealed she had experienced at least six other episodes of pneumonia since she was 4-year old, requiring several hospitalization and intravenous antibiotic therapy. Moreover, at the age of 3, she had developed synovitis of both wrists, ankles and knees, which was treated with oral glucocorticoids and with intraarticular injections of glucocorticoids with partial benefit. She also presented with stunted growth (-3 SD for height and weight) and a wobbly gait, consistent with the history of chronic arthritis and joint deformities (see supplementary material). She started walking at 19 months of life and never improved much from her initial wobbly gait. Walking difficulties had been reported by the age of 3, with some falls occurring occasionally. Pediatric sarcoidosis had been previously suspected because of the presence of inflammatory granuloma in the liver biopsy, performed because of persistently increased transaminase levels and mild periportal hyper echogenicity on ultrasound (US). Sarcoidosis was ruled out after detecting normal angiotensin converting enzyme and chitotriosidase blood levels. Based on the coexistence of arthritis and lung involvement, COatomer Protein subunit Alpha (COPA) syndrome was suspected [2]: however, rheumatoid factor (RF) and interferon signature (IS), were absent or normal, therefore excluding COPA. Taking into account the history of recurrent pneumonia with bronchiectasis, immunological examinations were performed to rule out an immunodeficiency. In addition, tuberculosis, cystic fibrosis and primary ciliary dyskinesia were considered as possible diagnoses and excluded after detecting normal QuantiFERON test, sweat test and bronchial brushing. In contrast, immunologic phenotyping, showed complete IgA and IgE deficiency, together with undetectable levels of antibodies in response to Pneumococcal, Tetanus Toxin and Hemophilus Influenzae B vaccines. Low numbers of B cells, CD4+/CD8 + T cells ratio and recent thymic emigrants (RTE) were found (1.4%), leading to the diagnosis of a combined T and B immunodeficiency (Table 1). Finally, taking into account the presence of the wobbly wide-based gait, previously attributed to chronic arthritis, together with the presence of granuloma in the liver serum alfa-fetoprotein (AFP) levels was measured, which resulted increased (276 ng/ml, normal value < 7 ng/ml). Neurological examination showed normal cranial nerves function, normal tendon reflexes and muscle strength; a subtle telekinetic tremor with a mild dysmetria were noted, so that actually a cerebellar involvement could be suspected (SARA score 7) [3]. The girl also presented bulbar telangiectasia (Fig. 3) and a diagnosis of A-T was made. This was subsequently confirmed by Whole Exome Sequencing that showed a pathogenic homozygous nonsense variant c.8831_8832del in the ATM gene (p.Leu2945ValfsTer10) [4]. Despite the lack of consanguinity, the parents came from the same village so that the presence of a genetic isolate could not be excluded. Finally, after pneumonia resolution oxygen supplementation was stopped; an adequate peak expiratory flow was measured, blood gases evaluation was normal and PEP-mask was prescribed at discharge because of the bronchiectasis, along with oral therapy with macrolides and a low daily dose of glucocorticoids [5].

**Table 1:** Immunological findings. Day 3 of presentation: the patient was febrile and with pneumonia

Immunoglobulin levels		*Normal values*
IgG	900 mg/dL	572–1474 mg/dl
IgA	**< 4 mg/dL**	34–305 mg/dl
IgM	241 mg/dL	31–210 mg/dl
IgE	0 UI/ml	0–90 UI/ml
White blood cells (%, n)	81,5% (28.620/mmc)	4.500–13.500
Neutrophil count (%, n)	11,8% (23.330/mmc)	1500–8000
Lymphocyte count (%, n)	3.380/mmc	1500–7000
B-cells (%, n)	**14.7% (497/mmc)**	14–30%
T-cells (%, n)	59.3% (2.004/mmc)	58–75%
CD4+ (%, n)	**15.8% (534/mmc)**	29–47%
CD8+ (%, n)	39.5% (1335/mmc)	17–33%
NK	23.9% (808/mmc)	4–17%
RTE (CD3 + CD4 + CD45RA + CD31+) (%, n)	**1.4% (8/mmc)**	38–66%
Vaccinal antibodies		*Protective*
Pneumococcal polysaccharide vaccine specific IgG	**< 3 mg/L**	> 35 mg/dl
Anti-Hemophilus IgG	**< 0.11 mg/l**	1–5 mg/l
Anti-Tetanus Toxin IgG	**0.45 UI/ml**	> 1 UI/ml
Anti-HbS antibodies	**0.1 mUI/ml**	> 5 mUI/ml

## Discussion and conclusions

Uncertain gait and immunodeficiency are both hallmarks of A-T. Neurological symptoms are usually the first to appear with an ataxic gait characterized by irregular foot placement, wide base, and instability. However, most children with classic A-T have a not progressing neurological picture for the first 4–5 years of life [1] and neuroimaging studies in the early childhood are normal [6], so that mild equilibrium abnormalities while walking in a straight line can be the only red flag. Oculomotor apraxia usually occurs later with nystagmus. Immunodeficiency usually appears with defective humoral immune response (hypogammaglobulinemia, predominantly low IgA, and low levels of antigen-specific antibodies to pneumococcal polysaccharide vaccine), low B cell numbers and impaired development of memory B cells. There can be also persistent T cell lymphopenia, with low numbers of CD4+, CD8 + T cells and RTE, reflecting thymus dysfunction in generating naive T cells [1]. The immunodeficiency pattern seen in A-T patients in the first 5 years of life is quite stable over time and will remain almost the same throughout their lifetime [7]. This immune dysregulation can lead to recurrent pneumonia, bronchiectasis, obliterative bronchiolitis and ILD, with about 25% of the patients developing chronic lung disease [1]. Oculocutaneous telangiectasia usually appears within 5–8 years but can also be absent, and, therefore, they are not mandatory for the diagnosis [8]. We believe that this case has some peculiarities that deserve to be described. In particular, the heterogeneity of manifestations secondary to the immune-dysregulation, as shown by early onset of polyarthritis, clinically indistinguishable from Juvenile Idiopathic Arthritis (JIA), and by the presence of liver granulomas, were misleading during the diagnostic approach, with consequent diagnostic delay [8]. Indeed, although rare, an autoinflammatory syndrome had been initially suspected because of the concomitant articular and lung disease.

The association of primary immunodeficiency syndromes with chronic arthritis has already been reported but appears to be rare. Cunningham-Rundles and Bodian firstly described 4 patients with common variable immunodeficiency (CVID) and chronic arthritis in their cohort of 248 patients ranging in age from 3 to 79 years [9]. Also, there are 3 reports in the literature dealing with noninfectious chronic arthritis in DNA repair defects: two of which in patients affected by Nijmegen breakage syndrome (NBS) and one in a young girl with atypical form of A-T [10–12]. Finally, Pasini et al. described the first case of polyarthritis occurring in a 15-year-old boy with typical A-T [13]. In our case arthritis onset occurred at the age of 3 years, resulting in joint loss of function. This initially led the treating physicians to consider polyarthritis and articular limitation as the cause of the uncertain gait. Arthritis secondary to immune-dysregulation seems to be clinically undistinguishable from JIA [13]. It can show variable positivity of autoantibodies but must be treated with caution because of the cancer susceptibility related to the use of common antirheumatic drugs (i.e. methotrexate). In this regard, lung Magnetic Resonance Imaging (MRI) and/or US should be performed in such patients for their radiological follow-up in order to minimize the cancer-related risk of ionizing radiation in DNA double-strand break repair (DSBR) diseases [14, 15].

Furthermore, a rare but well-recognized manifestation of immune deficiency in A-T is the formation of non-infectious cutaneous and visceral granulomas. This is due to abnormalities of the B and T cell compartments resulting in defective immune response to microbial antigens, as well as to inappropriate immune regulation and innate immune response-driven inflammation [16]. Our patient presented granulomatosis of the liver, but also a persistent rise in ALT and GGT levels with US finding of mild periportal hyper echogenicity. Progressive liver disease has been reported in patients with A-T especially after 12 years of age [17], with non-alcoholic fatty liver disease and fibrosis being the most frequent findings. GGT are the more sensitive marker of such condition [17]. It has been shown that progressive liver disease is concomitant to neurological deterioration and it is rarely present at earlier ages [18]. Therefore, periodic liver function monitoring should be performed in all patients with A-T.

The pathogenic variant found in this patient (c.8831_8832del) has been reported in few literature papers as associated with an absent or disrupted protein product secondary to a premature translational stop signal (p.Leu2945ValfsTer10) in the ATM gene. This can be responsible of the classical A-T phenotype but the severity of the clinical presentation can be very different depending on the residual activity of the phosphatidylinositol 3-kinase (PI3K) domain [1, 19]. Whenever suspected, AFP is the best diagnostic marker of A-T. AFP levels are very high in all newborns, and normally decrease to adult levels over the first year to 18 months. About 95% of people with A-T have elevated serum AFP levels after the age of 2 years, and levels of AFP appear to increase slowly over time [1, 20].

This case underlines the heterogeneity of A-T manifestations and the importance of always considering immunodeficiency when faced with a history of recurrent infections, even in the context of complex inflammatory phenotype. Children with recurrent pneumonia, and even more so if requiring hospitalization, should always be referred to an immunologist for a diagnostic evaluation. Arthritis and tissue granuloma present in this patient can be an epiphenomenon of the underlying immune-dysregulation. Although rare, A-T should be always ruled out in the presence of clinical presentation strongly suggestive of autoimmunity or autoinflammation, carefully searching for uncertain gait and/or bronchiectasis, even in the absence of bulbar or skin telangiectasia. When A-T is suspected, measurement of AFP represents the most useful diagnostic marker of the disease, which should then be genetically confirmed.

### Electronic supplementary material

Below is the link to the electronic supplementary material.

Supplementary Material 1

## Data Availability

not applicable.

## Abbreviations

AFP: Alpha-Fetoprotein
A-T: Ataxia-Telangiectasia
ALT: Alanine Aminotransferase
COPA: COatomer Protein subunit Alpha
CT: Computed Tomography
CVID: Common Variable Immunodeficiency
DSBR: Double-Strand Break Repair
GGT: γ-glutamyl transferase
ILD: Interstitial Lung Disease
IS: Interferon Signature
JIA: Juvenile Idiopathic Arthritis
MRI: Magnetic Resonance Imaging
NBS: Nijmegen breakage syndrome
PI3K: Phosphatidylinositol 3-Kinase
RF: Rheumatoid Factor
RTE: Recent Thymic Emigrants
WBC: White Blood Cells
WES: Whole Exome Sequencing
